# Delivery of sry1, but not sry2, to the kidney increases blood pressure and sns indices in normotensive wky rats

**DOI:** 10.1186/1472-6793-9-10

**Published:** 2009-06-05

**Authors:** Daniel Ely, Amy Milsted, Gail Dunphy, Shannon Boehme, Jeff Dunmire, Mike Hart, Jonathon Toot, Almir Martins, Monte Turner

**Affiliations:** 1 Department of Biology, University of Akron, Akron, OH 44325, USA

## Abstract

**Background:**

Our laboratory has shown that a locus on the SHR Y chromosome increases blood pressure (BP) in the SHR rat and in WKY rats with the SHR Y chromosome (SHR/y rat). A candidate for this Y chromosome hypertension locus is Sry, a gene that encodes a transcription factor responsible for testes determination. The SHR Y chromosome has six divergent Sry loci. The following study examined if exogenous *Sry1 *or Sry2 delivered to the kidney would elevate renal tyrosine hydroxylase, renal catecholamines, plasma catecholamines and telemetered BP over a 28 day period. We delivered 50 μg of either the expression construct Sry1/pcDNA 3.1, Sry2/pcDNA 3.1, or control vector into the medulla of the left kidney of normotensive WKY rats by electroporation. Weekly air stress was performed to determine BP responsiveness. Separate groups of animals were tested for renal function and plasma hormone patterns and pharmacological intervention using alpha adrenergic receptor blockade. Pre-surgery baseline and weekly blood samples were taken from *Sry1 *electroporated and control vector males for plasma renin, aldosterone, and corticosterone. BP was measured by telemetry and tyrosine hydroxylase and catecholamines by HPLC with electrochemical detection.

**Results:**

In the animals receiving the *Sry1 *plasmid there were significant increases after 21 days in resting plasma norepinephrine (NE, 27%) and renal tyrosine hydroxylase content (41%, p < .05) compared to controls. BP was higher in animals electroporated with *Sry1 *(143 mmHg, p < .05) compared to controls (125 mmHg) between 2–4 weeks. Also the pressor response to air stress was significantly elevated in males electroporated with *Sry1 *(41 mmHg) compared to controls (28 mmHg, p < .001). *Sry2 *did not elevate BP or SNS indices and further tests were not done. The hormone profiles for plasma renin, aldosterone, and corticosterone between electroporated *Sry1 *and control vector males showed no significant differences over the 28 day period. Alpha adrenergic receptor blockade prevented the air stress pressor response in both strains. Urinary dopamine significantly increased after 7 days post Sry electroporation.

**Conclusion:**

These results are consistent with a role for *Sry1 *in increasing BP by directly or indirectly activating renal sympathetic nervous system activity.

## Background

We have shown previously that there is a locus on the SHR Y chromosome that increased blood pressure (BP) in an additive manner by 20–25 mmHg and that The Y chromosome Sry locus increased tyrosine hydroxylase promoter activity [1-4] and increased several indices of sympathetic nervous system (SNS) activity [5]. We demonstrated that renal norepinephrine (NE) turnover rate is higher by 100% and renal NE content is 44% higher in males with the SHR Y chromosome [6]. Studies in our lab indicate that a candidate gene for this SNS and BP effect is the Sry gene complex on the rat Y chromosome. The Sry protein is a transcription factor that has been shown to be the testis determining factor in mice and humans [7], although Sry expression has been reported in adult testis, brain, adrenal gland and in additional tissues that have BP relevance in humans and rodents [8-11]. The Sry protein has potential target loci not involved in testis determination. We have demonstrated that one of the six copies of the Sry gene complex, *Sry1*, increased tyrosine hydroxylase (Th) promoter activity in transfected PC12 cells [7]. Th is the rate limiting enzyme in the catecholamine biosynthesis pathway; thus regulation of Th by *Sry1 *is a potential pathway for an Sry locus to affect BP. There were two components to the activation effects of *Sry1 *on the Th promoter, one is an indirect effect through an AP-1 transcription factor binding site. Consistent with this result is the observation that *Sry *binding sites have been identified in *Fra-1 *and *Fra-2*, both components of the AP-1 transcription factor [12]. Recently, we examined the impact of *Sry1 *expression in the adrenal medulla., *Sry1 *exogenously delivered to the adrenal medulla by electroporation increased adrenal Th activity, plasma NE and BP compared to vector controls [1]. In additional support of an *Sry *effect on Th, Dewing et al. has shown that *Sry *is expressed in the substantia nigra of both the mouse and rat brain and downregulation of *Sry *by antisense *Sry *in the substantia nigra produced a decrease in Th neurons and altered motor behavior [13].

We have also reported that *Sry *transcripts are present in the kidney of adult male rats [14]. Since the kidney is involved in several different mechanisms of hypertension, and based on our previous observations of SNS involvement in hypertension, we developed the following hypothesis: delivery of exogenous *Sry *to the kidney medulla of a normotensive WKY rat will increase BP through effects on renal Th activity, and elevated SNS indices.

The electroporation technique of gene delivery was developed primarily for use in cell culture, but it has also been successfully used in various tissues in the whole animal. For instance, delivery of exogenous genes produced physiological responses in nephrectomized rats, showing that rat erythropoietin injection corrected anemia [15], and raised hematocrit from 47% to 80% [16]. Tsuji et al examined the efficiency of intrarenal injection of DNA followed by in vivo electroporation and found that mesangial cells were transfected and no histological damage was observed [17].

## Methods

Adult WKY males (n = 82) were used in the following studies. The rationale for using WKY males is that they have normal blood pressure and if the exogenous Sry delivered would result in a BP elevation it would be better observed in the normotensive animal rather in one with borderline or fully developed hypertension. All animal protocols were approved by the Institutional Animal Care and Use Committee of the University of Akron. The experimental design explored four questions: 1. Does delivery of exogenous *Sry1, Sry2 *or control vector alter renal NE and BP?, 2. Does delivery of exogenous *Sry1, Sry2 *or control vector alter renal function or morphology?, 3. Does delivery of exogenous *Sry1 *or control vector alter plasma hormones involved in BP control-aldosterone, plasma renin activity and corticosterone?, and 4. Is the BP pressor effect blocked by an alpha adrenergic blocker?

The objective of the first experiment was to test the hypothesis that exogenous *Sry1 *would elevate BP, but *Sry2 *would not. *Sry1, Sry2 *or control vector was delivered to the kidney medulla of normotensive WKY rats (n = 6/group). Animals were implanted with telemetry probes (Data Sciences) in the abdominal aorta just above the renal artery as previously described (see below). The *Sry1 *expression construct, *Sry1*/pcDNA3.1(-), which includes the complete SHR *Sry1 *coding sequence (bp#11-520 of GenBank accession number, AF274872), as described previously.[7] The full length SHR *Sry2 *coding sequence, from an XbaI/Not l genomic fragment, was cloned into pcDNA3.1(-), to produce the expression construct, Sry2/pcDNA3.1(-) with the *Sry2 *coding region (bp#246-716 of GenBank accession number, AY157670).

The objective of the second experiment was to test the hypothesis that electroporation of *Sry1 *or empty vector to the kidney did not alter renal excretory function. Either empty vector or Sry1/pcDNA3.1(-)was injected into the left kidney of WKY rats (n = 6/group) following the same procedure as experiment 1. Twenty four hour urine and blood samples were analyzed for creatinine clearance, BUN and urinary protein, catecholamines and sodium, and histology to verify that the electroporation procedure did not cause renal damage. The same electroporation protocol was followed as in the first experiment. Adult male WKY were injected with Sry1/pcDNA3.1(-) (n = 6) or empty vector (n = 6) and electroporated to examine the tissue for damage. A control group of WKY kidneys (n = 6) that was not injected or electroporated was also examined for comparison.

The objective of the third experiment was to test the hypothesis that electroporation of *Sry1 *or empty vector did not alter plasma hormones (renin, aldosterone, and corticosterone) that potentially could be involved with high BP. The same *Sry1 *or control vector delivery protocol as above was used on WKY males (n = 4/group). Blood was collected weekly for 4 weeks before and after treatment under pentothal anesthesia (50 mg/kg, ip) and spun down for plasma separation, frozen at -80C and analyzed by RIA: plasma renin activity (Diasorin; Stillwater, MN), plasma corticosterone and aldosterone (Diagnostic Systems Laboratories, Inc. Webster, TX).

The objective of the 4th experiment was to examine stress induced telemetered BP with alpha adrenergic blockade (prazosin 0.5 mg/kg, ip.) and urinary catecholamines after *Sry1 *or control vector delivery to the kidney. The same *Sry1 *or empty vector delivery protocol as above was used on WKY males (n = 4/group).

### Electroporation

For all of the electroporation procedures the following protocol was used. Each tissue has different optimal electroporation parameters that have been described for gene delivery [18]. Based on previous research and our own pilot tests we performed electroporation in a pulsatile fashion immediately after DNA injection. Either 50 μg of Sry1/pcDNA3.1(-), or 50 μg of control pcDNA3.1(-) vector without *Sry1 *sequences was injected into the left kidney medulla of 6 WKY adult male rats using pentothal as an anesthetic (50 mg/kg, ip). *Sry1*, or control vector, was delivered by injection (28 g needle) followed by electroporation into the kidney medulla. A mark was placed on the needle to insure proper depth to the medulla and two injections were administered one to the upper quadrant and one to the lower quadrant of the kidney. A drop of glycerol was placed on the injection site before injection to prevent fluid backflow. Tweezer electrodes (BTX Tweezertrodes Model #522) connected to an electrostimulator (ElectroCellManipulator™, ECM^® ^830, BTX, a division of Genentronics), were placed on opposite sides of the kidney and 20 bipolar electrical pulses administered, 200 volts, each lasting 20 msec, at 1000 Hz after both injections. After stimulation the incision was closed and the animal allowed to recover. For the *Sry2 *studies the same procedure was followed but 50 μg of Sry2/pcDNA3.1(-), or 50 μg of control pcDNA3.1(-) vector without *Sry2 *sequences was injected into the left kidney medulla of WKY adult male rats(n = 6/group). These animals were also implanted with telemetry receivers and monitored as described below.

In order to verify that this procedure delivered *Sry1 *to the kidney, two WKY females were electroporated with *Sry1 *delivered to the kidney using the same protocol as described above. The plasmid-Sry1, cannot be distinguished from endogenous *Sry*, therefore the use of females for verification. Total DNA was screened for the presence of *Sry1 *by gel-based PCR using the primer set M1-P1 which amplify from the HMG box of all Sry loci.

### Blood Pressure, Heart Rate and Alpha Blocker Studies

Blood pressure and heart rate were measured continuously in conscious animals by telemetry and plotted weekly before and after electroporation (Data Sciences) [5]. For the stress procedures, air stress was performed by using compressed air which traveled through Tygon tubing (1/4 × 1/16 inch) connected to a Pasteur pipette and the air was directed into the face of the rat at a pressure of 10 psi for 30 seconds. During this time three 10 second pressure readings were taken and the highest of the three used for the air stress results. The air stress was performed on all the rats before *Sry1*or *Sry2 *or empty vector was delivered into the left kidneys for the baseline values. After *Sry1 *or *Sry2 *or empty vector delivery, air stresses were done at days 8, 15, and 22. In another group of adult WKY males *Sry1 *(n = 4) or empty vector control (n = 4) was delivered to the kidney in the same manner as above. Day 22 post Sry or EV delivery 0.5 mg/kg prazosin was injected (ip) and after 30 min an air stress test performed (30 sec).

### Catecholamine and Tyrosine Hydroxylase Analysis

Levels of renal Th and catecholamines were measured 21 days after electroporation. Th and catecholamines were measured by HPLC with electrochemical detection as we previously reported [1] (Waters 2465, Milford, MA). For Th analysis kidneys were homogenized in 500 μl of 0.25 M sucrose. The homogenate (100 μl) was added to both a blank tube and a reaction tube. The chemical reaction was based on procedures developed by Nagatsu [19] and modified by Hooper et al [20] and Kumai et al [21] followed by extraction using the same method as for catecholamines.

### Urine Collection and Analysis

For the urine collection studies rats were placed into metabolic cages for 24 hour urine collection during baseline before plasmid delivery and during various days post plasmid delivery. They were given Pro-lab 3000 food and water ad libitum. Mineral oil was placed into the urine collection cup to prevent urine evaporation over the collection period for sodium and creatinine measures. For the catecholamine collections 1 ml of 6N HCl and mineral oil was added to the collection cup. The 24 hour urines were tested for total protein by sulfosalycidic acid turbidity method and creatinine clearance (Thermo Electron; Pittsburgh, PA) [22] and sodium by flame photometry. Urinary catecholamines (24 hr) were performed at baseline, stress, 7,14, 21, and 28 days post electroporation. Using a separate group of animals (Vector control, n = 8 and Sry1, n = 8) the same procedure was followed as in experiment 2 and urine was collected at 7, 14 and 21 days for sodium and catecholamines. At day 14 and 21 a non-selective dopamine blocker, metoclopramine was injected (0.5 mg/kg, ip) to each animal and 24 hour urine collected.

### Kidney Morphology

In the third study the electroporated left kidney was removed after 21 days, stored in 10% buffered formalin, dehydrated in a tissue processor (Tissue-Tek, Miles Inc., Elkhart, IN) and paraffin embedded (Paraplast Plus, Fisher Sci). Sections were cut at 6 μ and stained with hemotoxylin and eosin (Richard Allan Scientific, Kalamazoo, MI) to assess the tissue for potential gene delivery-electroporation injury. Glomeruli and tubules were scored blindly by 3 individuals for pathological changes [23].

### Statistical Analysis

BP, urinary Na, hormones, and urinary catecholamines were tested with 2-way ANOVA(group and time) with repeated measures. Th, creatinine, BUN, protein and catecholamine renal content were analyzed using t-tests and; means and standard error of the means are reported; significance was assumed if p < .05.

## Results

**Figure 1:**
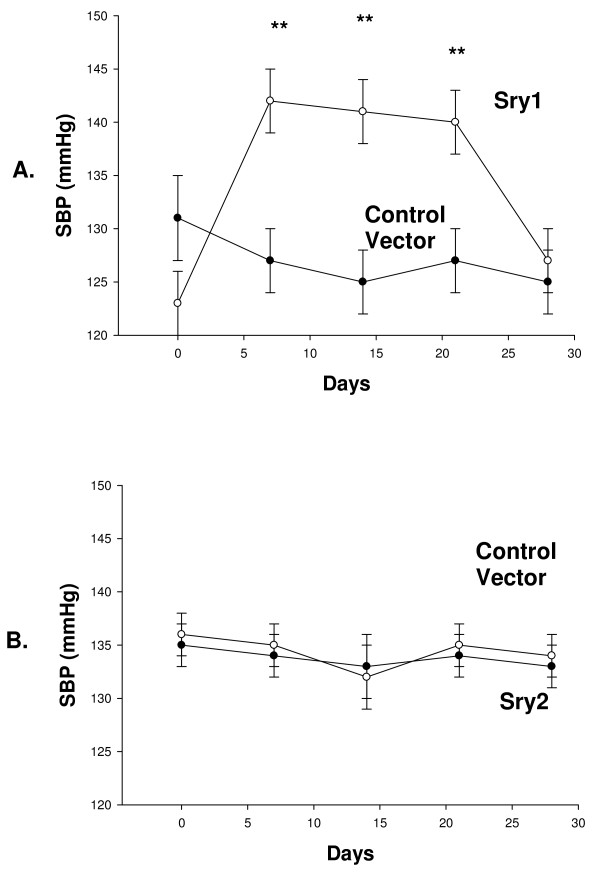
A. Telemetered systolic blood pressure (SBP) was significantly elevated after Sry1 delivery to the kidney as compared to control vector (means, +/- s.e.m., ** = p < .01, n = 6/group). B. Telemetered systolic blood pressure (SBP) was not significantly elevated after Sry2 delivery to the kidney as compared to vector controls (means, +/- s.e.m., no significant differences).

**Figure 2:**
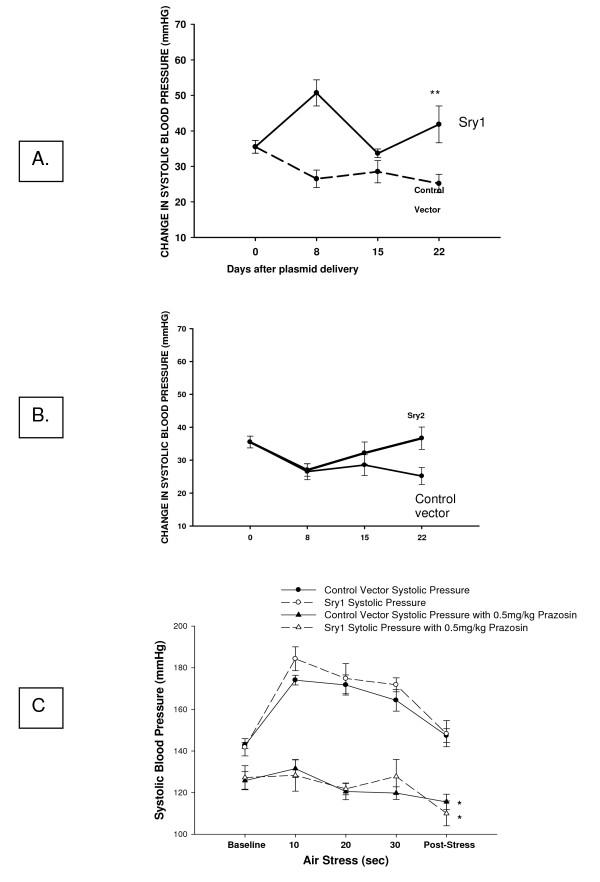
A. Change in telemetered systolic blood pressure (SBP) from baseline during 30 sec. air stress in Sry1 and control vector groups (means, +/- s.e.m., 2-way ANOVA, ** = p < .01, n = 6/group). B. Change in telemetered systolic blood pressure (SBP) from baseline during 30 sec. air stress in Sry2 and control vector groups (means, +/- s.e.m., n.s., n = 6/group). C. Alpha blockade with prazosin (0.5 mg/kg, ip) effectively blocked the stress induced BP rise, means, +/- s.e.m., < .05.

**Figure 3:**
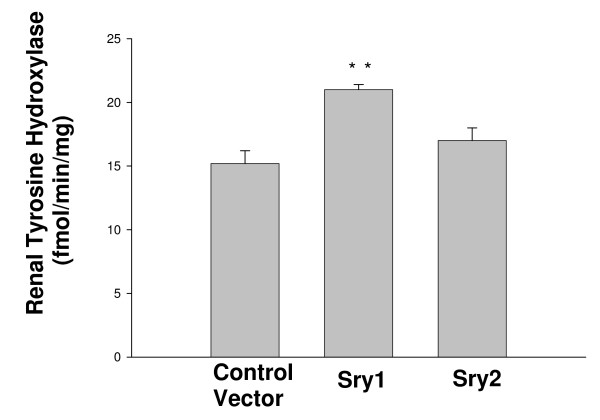
Renal tyrosine hydroxylase activity (measured as L-DOPA formed per minute per mg tissue, fm/min/mg) in kidney 21 days after plasmid delivery to Sry1 (n = 6), Sry2 (n = 4) and control vector (n = 6) (means, +/- s.e.m., ** = p < .01).

**Figure 4:**
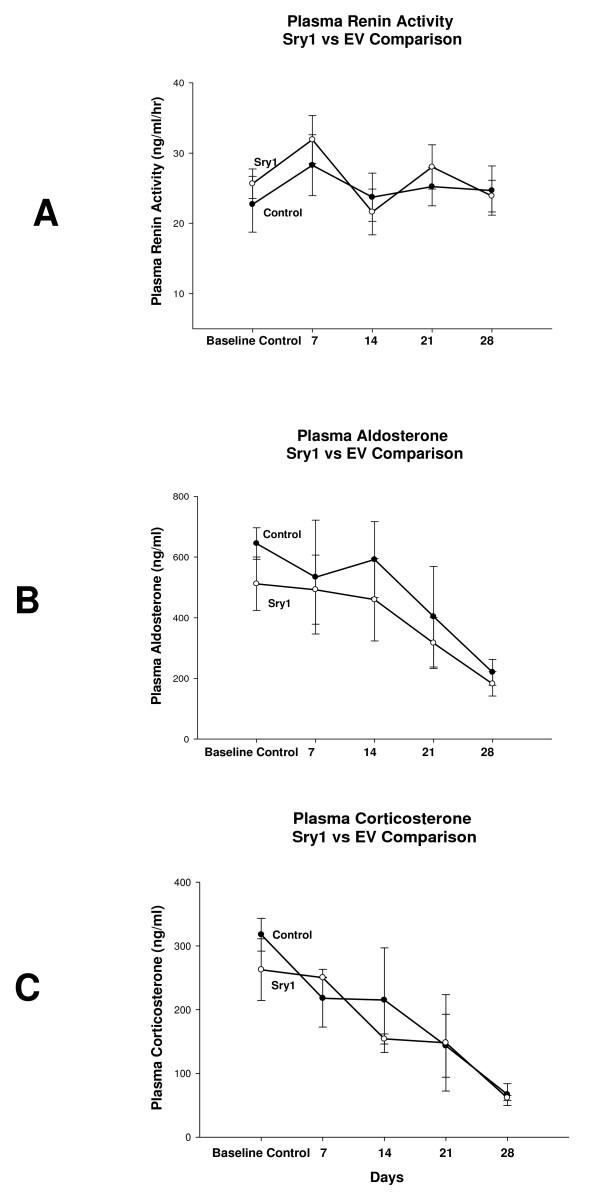
**Hormone profiles over time in control (solid circles, n = 4) and Sry1 (open circles, n = 4) animals for A. plasma renin activity, B. plasma aldosterone, and C. plasma corticosterone (means, +/- s.e.m., no significant differences)**.

**Figure 5:**
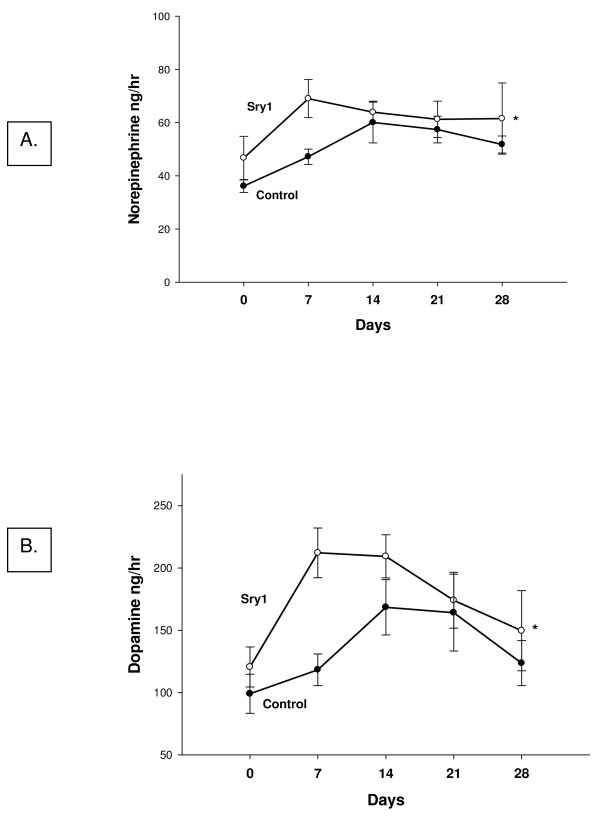
Urinary catecholamines (A. norepinephrine, B. dopamine) in animals from experiment 4 from across time in *Sry1 *or control vector, n = 6–7/group), means, +/- s.e.m.,*= p < .05, ** = p < .01 compared to control vector.

Figure 1A shows BP before and after delivery of either *Sry1*, or the empty control vector to the kidney. There was a significant increase in BP after *Sry1 *delivery compared to control vector at days 7,14, 21: 143,142,141 vs. 127,125,127 mmHg, respectively (p < .05) and BP was normalized after 28 days. When *Sry2 *was delivered to the kidney in the same manner there was not a BP effect (Figure 1B). Figure 2A shows the significant and consistent elevation in pressor response to air stress in the *Sry1 *group (41 mmHg) compared to controls (28 mmHg) at days 8 and 22. The drop in pressor response that occurred at day 15 is unexplained. When *Sry2 *was delivered to the kidney in the same way there was no difference in BP response to air stress between *Sry2 *and controls (Figure 2B). Figure 2C shows that the pressor response to air stress was completely blocked in both Sry1 and control animals treated animals with prazosin. The BP responses cannot be explained by heart rate which were the same between stressed *Sry1*(333 +/- 32) and control groups (331 +/- 7). Figure 3 shows that 21 days after *Sry1 *delivery to the kidney, Th was significantly increased compared to the vector control (21 vs. 15 fmol/min/mg, p < .05), however *Sry2 *did not produce any significant Th difference compared to controls. With regard to kidney catecholamine content there were no significant differences in NE or dopamine content between animals delivered vector or *Sry1 or Sry2 *DNA measured at 21 days. Creatinine clearance, urinary protein, sodium and BUN were not significantly different between groups and were within normal range, suggesting that the electroporation procedure did not damage the kidney or alter renal function (Table 1). Urinary Na was also measured at 7, 14 and 28 days after electroporation besides the 21 value reported in Table 1 and was not significantly different between groups (Control vector vs. Sry1: 7 day = 0.11 vs. 0.13 mmol/hr/100 gms, 14 day = 0.15 vs. 0.14, 28 day = 0.18 vs. 0.17). Plasma NE was significantly elevated (57%) 21 days after Sry1 delivery compared to vector control (Table 1). Figure 4 shows the pre-surgery baseline and weekly plasma levels of renin, aldosterone, and corticosterone for the *Sry1 *and control vector groups. There were no significant differences between treatment groups for each plasma hormone at all time periods sampled. Figure 5 shows that 7 days after Sry1 delivery urinary dopamine and NE were significantly elevated but returned to baseline levels with time (two-way ANOVA, strain difference, F = 5.1, p < .05 for dopamine and F = 4.6, p < .05 for NE).

**Table 1:** Urine and Plasma Profile

	Urinary Protein (mg/dl)	BUN (mg/dl)	Urinary Creatinine Clearance (ml/min/gm bw)	Urinary Na (mmol/hr/100 gms)	Urinary Na With Dopamine Blockade(mmol/hr/100 gms)	Plasma NE (pg/ml)	Plasma Epi (pg/ml)
Control Vector (n = 6–7)	46 *+/- 15*	13.6 *+/- 0.8*	0.65 *+/- .07*	0.17 *+/- .01*	0.11 *+/- .013*	375 *+/- 70*	95 *+/- 10*
Sry1 (n = 6–7)	53 *+/- 15*	15.4 *+/- 0.2*	0.63 *+/- .09*	0.17 *+/- .01*	0.15 * +/- .011	590 * +/- 20	80 *+/- 5*

Means, +/- s.e.m., * = p < .05 compared to control vector. Data collected at 21 days.

After 21–28 days all catecholamine levels were not different from baseline values.

**Figure 6:**
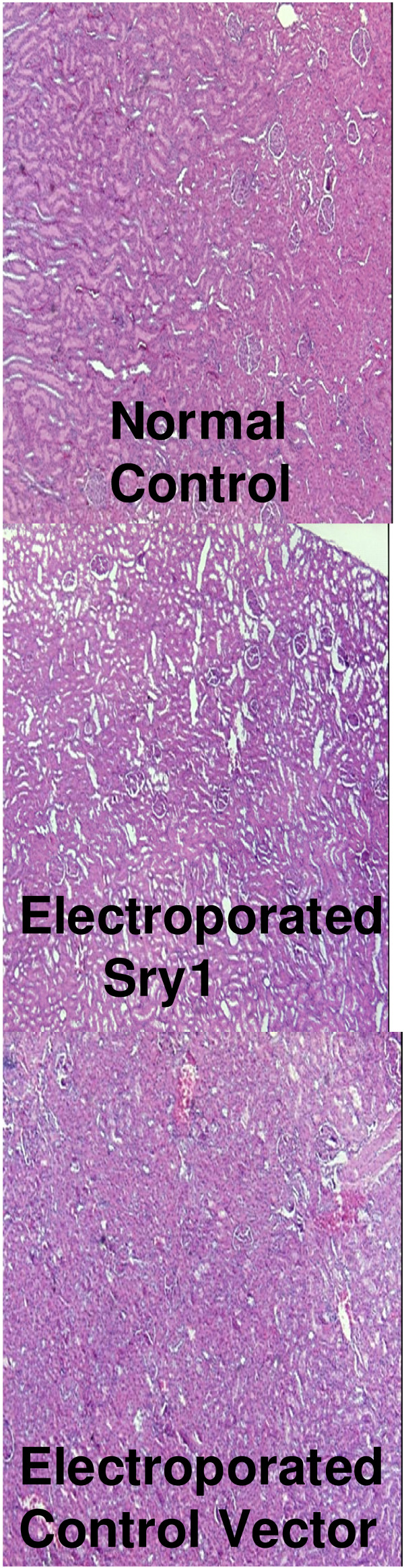
**Representative kidney sections (6 μ) stained with H&E to show glomeruli and tubules in: non-electroporated controls, Sry1 electroporated, and control vector electroporated animals**.

Histological examination of successive sections through the kidney medulla did not show any morphological differences between non-electroporated controls, control vector, or *Sry1 *delivered (Figure 6).

The females that had *Sry1 *electroporated in the left kidney were positive for *Sry *in the left kidney after 8 days, they were negative in the right kidney and positive in the liver. After 21 days the left and right kidney were negative but the liver remained positive.

## Discussion

Since the Y chromosome is inherited as a unit, determining which gene or genes are responsible for the hypertensive effect of the SHR Y chromosome requires the testing of potential Y chromosome hypertension genes. We have proposed *Sry1 *as a potential candidate gene for the Y chromosome hypertension. One step in this process is demonstrating that *Sry1 *can affect BP [24], but there are six *Sry *loci on the SHR Y chromosome [11]. Whether the effects of the hypertensive Y chromosome are unique to a single locus, *Sry1*, or a combination of more than one of the *Sry *loci is not known [11]. In this investigation we have electroporated *Sry1 *and *Sry2 *into the normotensive WKY kidney. *Sry1 *has been demonstrated to have a BP effect when exogenously expressed in the adrenal [1]. *Sry2 *is the most divergent of the 6 Sry loci [11] and thus the most likely to have a different effect.

Our main finding was that *Sry1 *delivered to the kidney resulted in elevated BP while *Sry2 *did not. The time scale of the BP response is consistent with the responses of other genes delivered by electroporation which is about 7–21 days after electroporation for peak effect [1,24]. The lack of a BP increase with control vector or *Sry2 *demonstrates that the BP increase was due specifically to the addition of *Sry1 *sequences, not to electroporation itself or an effect of the plasmid sequences. The divergence of *Sry2 *has apparently changed the binding or transcriptional activation characteristics of the *Sry2 *protein. The difference between *Sry1 *and *Sry2 *has to be the result of differences inherent in the proteins and not a difference in transcription of the *Sry1 *and *Sry2 *plasmid since both have the same promoter and plasmid. Whether or not the other Sry loci have hypertensive potential like *Sry1 *must to be tested individually as each locus has unique differences.

The electroporation of exogenous *Sry1 *DNA in the kidney of WKY rats increased BP by 15 mmHg, comparable to the 20 mmHg effect produced through breeding by replacing the entire SHR or WKY Y chromosome. We saw a similar BP increase when *Sry1 *was electroporated into the adrenal gland [1]. These increases are larger or equal to the effects of BP QTLs in other rat models of hypertension [25], which are potentially composed of multiple genetic BP components. Our results confirm the potential physiological influence of *Sry1 *on BP.

The demonstration of an *in vivo *effect of *Sry1 *in the kidney is of physiological consequence only if *Sry1 *is normally expressed in the SHR kidney. We have previously demonstrated the presence of *Sry *transcripts in the kidney without regard to locus. Although all six *Sry *loci are expressed in the 15 week old adult male testis, the adrenal and testis have a different expression pattern of the *Sry*; *Sry2 *is the primary transcript in the adrenal but significantly reduced in the testis [11]. The expression pattern of *Sry1 *or the other copies in the kidney or at other developmental times in the adrenal has not yet been reported.

Based on two of our previous studies, the SNS was indicated as involved in the elevated BP mechanism: 1) in PC12 cells [7]*Sry1 *increased Th promoter activity by 50% compared to control vector, and 2) we found a 49% increase in Th in the rat adrenal medulla [1] after *Sry1 *electroporation and similar effects on BP. We have not yet electroporated both tissues (adrenal gland and kidney) in a single animal to see if there is an additive effect. The demonstration of a BP increase with electroporation of *Sry1 *into either the kidney or adrenal gland indicates that *Sry1 *could have its effect in either organ or in both. The lack of effect of *Sry2 *on BP or SNS indices further supports our hypothesis that it is specifically *Sry1 *that produces the elevated BP. This is not to preclude other phenotypic effects of *Sry2*. A question that arise is the possibility that by injecting *Sry1 *into the upper and lower pole of the kidney, some *Sry1 *from the upper site may overflow into the left adrenal gland and influence the production of adrenal NE which could then increase plasma catecholamines and increase BP. To check this possibility we measured left adrenal NE and E content in the animals from the hormone study and found there were no significant adrenal gland differences in NE and epinephrine content between renal delivered *Sry1 *or control vector (data not shown). This does not exclude the possibility that some *Sry1 *plasmid may spill over to the circulation and increase *Sry1 *levels in other tissues. In females who lack the Sry gene, we can identify the *Sry1 *plasmid delivered. Detection of it in the kidney shows that it was present after 8 days, but also some via the circulation was found in the liver at 8 and 21 days. This suggests that it could be transported to other tissues and be expressed. Further studies are documenting tissue distribution and cellular localization.

With regard to the general concept of the involvement of the renal SNS and hypertension, it has been shown that increased renal sympathetic nerve activity has diverse functions in regulating the intrarenal effectors, such as the tubules, the blood vessels, and the juxtaglomerular granular cells. In a review of renal neural regulation by Di Bona [26], the SNS is shown to coordinate the circulatory, filtration, reabsorptive, excretory, and renin secretory contributions in the kidney. The SNS appears to be able to affect vascular, tubule and JG cell function individually, as well as in concert [26]. The concept of functionally specific renal sympathetic nerve fibers allows more finite control of renal function. In addition, the idea that *Sry *may be functionally active in the kidney is one more example of how the SNS can differentially provide fine tuning of organ function. In the SHR model of hypertension the SNS has been shown to play an important role in the development of BP control and in hypertension [27]. The SNS is the primary mediator of acute changes in BP and also chronically contributes to BP regulation.

The evidence supporting an *Sry1*-SNS interaction is mounting. SHR/y males have more renal NE content and a 67% increase in renal NE turnover compared to WKY males, indicating greater SNS activation attributed to the SHR Y chromosome [6]. In fact, the kidney NE turnover rate in the SHR/y males was the same as in SHR males, suggesting that the catecholamine pathway, at least in the kidney, is significantly influenced by the Y chromosome [5]. Also the pressor response to air stress appears to be mediated by the SNS and was on the average, 15 mmHg higher in the presence of exogenous *Sry1 *compared to control vector. Alpha adrenergic receptor blockade eliminated the pressor response in both strains suggesting that the pressor response was due to SNS activated vasoconstriction. Consistent with the specificity of an effect for *Sry1*was the lack of SNS responses after administration of *Sry2*.

In addition, we expected to observe that *Sry1 *would cause a renal increase of the rate limiting enzyme for NE synthesis, Th, to be elevated. Indeed renal Th was elevated after *Sry1 *but not after *Sry2 *delivery. Elevated Th and/or catecholamine levels are established physiological characteristics of many hypertensive animal models and human studies [26-36]. We have shown that the Y chromosome from a SHR male when backcrossed to a normotensive WKY female increased SNS indices [4] and maintained an increase in BP of about 20 mmHg even after 11 generations of backcrossing sons to WKY females in the SHR/y strain [3].

What are the pathways whereby Sry can lead to elevated Th? One possibility is that the Sry protein travels to the celiac ganglia where the cell bodies that contain Th are located. Recently we have verified that endogenous Sry is present in celiac ganglia. Based upon our *in vitro *studies, *Sry1 *promotes Th transcription [7]. Th may travel down the sympathetic nerves to the kidney residing in the nerve terminal and some Th may be also released into the synapse. We do know that in whole kidney there is Th activity, and when blood is removed from the kidney by saline perfusion, the total Th content does not change, so the Th measured is content from the kidney tissue and not the blood. Th may also be synthesized or released from the kidney itself. For example, in cultured rat mesangial cells, both Th and NE are present suggesting that the synthetic materials are present for the catecholamine pathway [37]. We assume this is also true in the whole animal, i.e. renal synthesis of NE.

Besides elevated renal Th we expected to observe increases in other SNS indicators: renal NE content and urinary catecholamines in the *Sry1 *treated animals. Although, renal Th at 21 days and urinary catecholamines (7 days) were elevated, renal NE content only showed an elevated non-significant trend. The renal NE content reflects a chronic measure of total stores of NE over time. Since renal Th was elevated we expected to observe renal NE content to be elevated. It may be that since the renal content is so high (80–90,000 pg/gm in a 1 gm kidney) that the *Sry1 *induced differences are small enough they are not reflected in total content differences. Multiple and complex mechanisms contribute to Th activity and catecholamine synthesis during SNS activation. Short term mechanisms include feedback inhibition and enzyme phosphorylation. Long term mechanisms include changes in Th synthesis [34]. There appears to be an uncoupling of the tissue NE content, activity of Th and the release of NE. Two storage pools of NE exist in both the SNS nerve terminals and in the adrenal medulla: a small readily releasable pool of newly synthesized NE and a large reserve pool in long term storage [30]. For example, studies in rats show a 35% reduction in Th activity at all ages studied (5, 12, 22 weeks) while plasma NE was 3–4× higher in SHR compared to WKY rats [36]. Therefore, NE storage, cytoplasmic pools and plasma levels need not be in a linear direct relationship.

The counter balance to renal SNS-NE effects and sodium retention is the effect of renal dopamine as a natriuretic factor. Renal dopamine does not derive from the SNS or the typical neuronal NE pathway but instead proximal tubule cells use circulating or filtered L-DOPA as a source of dopamine in a paracrine fashion [38]. So the urinary excretion of dopamine reflects the renal production of the amine. The dopamine receptors in the kidney are D1 and D2 types in brush-border and basolateral membranes of proximal tubules and can alter blood flow and sodium excretion [39]. With regard to differences between SHR and WKY males, SHR males excretes more dopamine and NE than WKY males across varying sodium intakes [40] even though the dopamine receptor density is not different [41]. If the Y chromosome and Sry were involved with this mechanism, we would expect to see potentially more urinary dopamine excreted in the *Sry1 *animals. Indeed, after 7 days there was almost a doubling of urinary dopamine in *Sry1 *animals compared to vector controls. Consequently urinary sodium excretion may have been expected to increase due to a dopamine natriuretic effect but there was no difference in sodium excretion at 7, 14 or 21 days. There is a strong possibility that the elevated SNS activity reflected as increased plasma and urinary NE throughout this period, may have normalized the sodium excretion. It has been shown that increased renal SNS activity increases renin secretion rate and decreases urinary sodium excretion [26]. The dopamine blockade experiment suggested that even though there was no difference in sodium excretion at 7 days with no blocker, when the dopamine blocker was given at 14 days the *Sry1 *animals did not respond with a significant decrease in sodium excretion as expected, whereas the vector controls had a significant 34% decrease. We do not know at this time if Sry delivery altered dopamine receptor density or if that the initial effect on dopamine (83% elevation) was so large that the dopamine blocker was not enough to reduce the sodium excretion to values seen in the vector controls.

Other potential mechanisms that could elevate BP in response to *Sry1 *are some of the hormones involved in BP regulation. The lack of plasma renin activity difference between *Sry1 *and control vector, imply that *Sry1 *is not acting via the circulating renin angiotensin system (RAS), but does not eliminate effects of the renal RAS. In addition, analysis of plasma aldosterone showed no significant differences between controls and *Sry1*, suggesting that sodium retention via aldosterone regulation is not a key player in the noted BP increase. As with plasma renin and aldosterone, plasma corticosterone showed no significant differences between controls and *Sry1 *animals for each time period sampled, further indicating that increased basal corticosterone is not responsible for the BP differences. It was curious, however, that both aldosterone and corticosterone levels were elevated at the beginning of the study (baseline and 7 days) and continued to decrease toward more normal levels with time. This possibly may be due to a stress effect of getting use to the blood collection procedure. Therefore, the similarities in these hormone profiles between *Sry1 *and control vector males indicate that the increased BP following renal transfection of *Sry1 *is not via modulation of circulating renin, aldosterone, or corticosterone.

## Conclusion

Our results support the hypothesis that *Sry1 *but not *Sry2 *from the SHR male increases kidney Th, and elevated SNS markers and increased peripheral vasoconstriction resulting in increased BP in a WKY normotensive animal. The findings presented here confirm and extend the argument for *Sry1 *as at least one of the genes responsible for the hypertensive Y chromosome phenotype. These results are consistent with an increased SNS activity mechanism, but does not preclude Sry effects on other important blood pressure regulation systems like the tissue renin angiotensin system. *Sry1 *is the first Y chromosome locus to have hypertensive potential confirmed; however, this result is not sufficient to conclude that *Sry1 *is solely or directly responsible for the hypertensive effect of the SHR Y chromosome, however not all Sry loci raise BP as seen by the lack of a BP effect with the electroporation of *Sry2 *to the kidney. The *Sry1 *sequence and expression need to be compared from normotensive and hypertensive Y chromosomes and differences consistent with the electroporation results demonstrated to conclusively show that *Sry1 *is responsible.

## Competing interests

The authors declare that they have no competing interests.

## Authors' contributions

AM provided plasmid and Sry harvesting and participated in design of study and edited the manuscript. GD spearheaded the assays and provided histological expertise and telemetry data. SB provided assay expertise and telemetry skills. JD assisted with electroporation and molecular techniques. MH performed the kidney function studies. JT assisted with electroporation procedure, data analysis and editing the manuscript. MT helped to write and edit the manuscript. DE is senior investigator, designed the study and was the primary writer of manuscript.
